# Food-Specific Inhibition Training for Food Devaluation: A Meta-Analysis

**DOI:** 10.3390/nu14071363

**Published:** 2022-03-24

**Authors:** Yingkai Yang, Le Qi, Filip Morys, Qian Wu, Hong Chen

**Affiliations:** 1 Faculty of Psychology, Southwest University, No. 2 Tiansheng Street, Beibei District, Chongqing 400715, China; 2 School of General Education, Chongqing City Management College, Chongqing 401331, China; zjs852314577@email.swu.edu.cn; 3 Montreal Neurological Institute, McGill University, Montreal, QC H3A 2B4, Canada; filip.morys@mail.mcgill.ca; 4 The Lab of Mental Health and Social Adaptation, Faculty of Psychology, Research Center of Mental Health Education, Southwest University, Chongqing 400715, China; chelle@email.swu.edu.cn; 5 Key Laboratory of Cognition and Personality (Ministry of Education), Southwest University, Chongqing 400715, China

**Keywords:** food-specific inhibition training, food devaluation, meta-analysis

## Abstract

Theories have suggested that food-specific inhibition training could lead to food devaluation which, in turn, may help people to regulate their eating behavior. In this review, we have synthesized the current literature on this topic by conducting a meta-analysis of studies investigating the effects of food-specific inhibition training on food evaluation. We identified 24 studies—with 36 independent samples, 77 effect sizes, and 3032 participants—that met our inclusion criteria. Effect sizes were analyzed using the robust variance estimation in random effects meta-regression technique. The results indicate that food-specific inhibition training can lead to statistically significant reductions in food evaluation. More specifically, it was observed that the effects of training on participants’ food evaluation differed according to the type of evaluation; food-specific inhibition training significantly decreased participants’ explicit food evaluation, but not their implicit food evaluation. However, because most of the included studies focused on trained food items and short-term outcomes in normal-weight samples, more research is needed on the continuance of the training effects, as well as on the extent to which effects can be generalized to untrained food items or different populations (e.g., overweight or obese individuals).

## 1. Introduction

Eating behaviors can be defined as the internal driving force for the approach and ingestion of food [1]. Dysfunctional eating behaviors, such as overeating and binge eating, can be aggravated by many factors, including higher evaluations (e.g., greater craving or wanting) of high-energy-density foods (e.g., foods that contain large amounts of sugar and fat) [2]. A recent systematic review proposed that both external (e.g., obesogenic environment) and internal (e.g., anxious/depressive states, impulsivity) factors could contribute to the high evaluations of high-calorie foods [3]. Critically, according to the incentive sensitization theory [4] and the dynamic vulnerability model of obesity [5,6], increased incentive salience of high-calorie food cues and the activity of the brain’s reward system in response to high-calorie food cues can predict overeating and weight gain. This prompts us to ask the question: how can we reduce the reward responses or evaluations of such foods?

### 1.1. Food-Specific Inhibition Training

One way of devaluing appetitive foods is via food-specific inhibition training [7,8]. Inhibition or inhibitory control is defined as the ability of an individual to inhibit their impulses and habitual or dominant behavioral responses to stimuli in order to select a more appropriate behavior that is consistent with completing their goals [9], and this is a key component of broader constructs such as executive function and self-regulation [10,11]. Two types of inhibition training have thus far been developed, namely, general inhibition training, and food-specific inhibition training [12,13]. The aim of general inhibition training is to increase overall inhibitory control through responses to often-arbitrary cues. Food-specific inhibition training, by contrast, pairs specific health-related cues (e.g., high-calorie food cues) with “no-go” or “stop” signals to promote associative links between such cues and the engagement of inhibitory control. For example, during food-specific inhibition training using a food go/no-go task, participants need to quickly respond (e.g., press button B) to the food picture (e.g., high- and low-calorie food pictures) displayed on the computer screen, and to withhold this response when a stop signal (e.g., the frame around the picture turning bold) is displayed. Critically, in a task aimed specifically at retraining particular behaviors (e.g., responses to high-calorie food cues), the no-go cue is disproportionately paired with high-calorie food items (e.g., 100%). In a control task, however, go and no-go cues are usually paired equally with non-food items [14].

Regarding general inhibition training, findings from several studies have demonstrated that this type of training is incapable of changing unhealthy eating habits [15,16]. Unsurprisingly, to the best of our knowledge, no further studies have been conducted to determine whether general inhibition training could influence food evaluation. By contrast, numerous studies have been carried out examining the effects of food-specific inhibition training on reducing the consumption of high-calorie foods [17,18,19,20]. Importantly, meta-analysis and systematic reviews have confirmed this training to have a small-to-moderate effect [12,21,22] (for relevant *p*-curve analyses, please see [23,24,25]). Furthermore, several theoretical accounts have been presented to explain how food-specific inhibition training changes eating behaviors [7,26,27,28]. One of the most likely explanations is that food-specific inhibition training could work through food devaluation [7,8].

### 1.2. Food-Specific Inhibition Training and Food Devaluation

Several explanations for this devaluation effect have been offered. Firstly, the behavior–stimulus interaction (BSI) theory [26,28] postulates that rewarding stimuli trigger strong approach reactions, which need to be inhibited when the stimuli are paired with a no-go/stop cue. Furthermore, in order to reconcile the conflict between the approach tendency elicited by motivational stimuli and the need to inhibit this tendency, a negative affect elicited by the conflict [29] is then attached to the stimuli, meaning that the evaluation of these stimuli is decreased in order to facilitate subsequent response [30].

Another possible explanation for this devaluation is that repeated inhibition toward specific stimuli (e.g., high-calorie food cues) during inhibition training may create automatic stimulus–stop associations [27]. Furthermore, associative learning theories have argued that action and valence are closely coupled, such that stopping is associated with punishment, whereas going is associated with reward [31,32,33]. It is therefore plausible that no-go/stop foods become increasingly disliked via their associations with automatic response inhibition [34,35].

In addition, several researchers have argued that rapid successful motor inhibition could have suppressive effects—not just on a motor level, but also on cognition [36]—which could impact value [37]. More specifically, it is believed that rapid action stopping may occupy working memory capacity [38,39], which then leads to less accurate representations of the no-go stimuli and, in turn, lower evaluation.

### 1.3. Potential Moderators of Training Effects

Although, as outlined above, food-specific inhibitory control training could lead to food devaluation, there have been some inconsistencies in the related literature. In order to understand these inconsistencies, it is important to examine the empirical literature so as to identify the factors that have been suggested as potential moderators of the training effects.

The type of evaluation may be an important moderator. Researchers in this field have typically assessed two types of food evaluation: the implicit evaluation, which is measured using the implicit association task or the affective priming paradigm, and the explicit evaluation, which is measured by a visual analogue scale (VAS) (e.g., “How attractive does this food item look to you?”). It should be noted that when using a VAS to measure explicit food evaluation, the researchers’ areas of interest differ. For example, the VAS could be used to measure food preference, craving, palatability, attractiveness, monetary value, etc. Some studies have shown that not responding to food items in food-specific inhibition training may lower the explicit evaluations of these items [26,40]. In contrast, evidence of the effect of food-specific inhibition training on implicit food evaluation is relatively sparse [41,42].

Another important moderator of training effects on food evaluation may be the training paradigm. Researchers have speculated that a higher possibility of food inhibition in the go/no-go task compared to the stop-signal task might lead to a greater degree of effectiveness in terms of changing eating behaviors and food devaluation [12,22].

Food novelty in the evaluation tasks may also be an important moderator. Indeed, some studies have suggested that the devaluation effect is specific to the trained food cues, and cannot be generalized (e.g., new stimuli) [18,26].

Body weight (e.g., excessive weight/obesity vs normal weight) may be another important moderator, for two reasons [35]: (1) overweight and obese individuals may gain more from the training compared with normal-weight participants, since they could have lower inhibition capacity [43]; and (2) the greater responsivity to food of overweight or obese participants [44] may impair their performance in the inhibition training, rendering the training less efficient.

Finally, we conducted exploratory analysis to explore the moderating roles of age, sex, and length of follow-up.

### 1.4. The Meta-Analysis

To the best of our knowledge, no previous meta-analysis has been conducted on this topic. In this study, therefore, we conducted the first quantitative meta-analysis of existing studies examining the effects of food-specific inhibition training on food evaluation. Such an analysis is important, since it allows for a range of effect sizes across studies, and provides a more powerful estimate of true training effects. Furthermore, we conducted moderator analysis to determine whether the aforementioned potential moderators account for unique variance in the effects of food-specific inhibition training across studies.

## 2. Method

### 2.1. Study Selection and Inclusion Criteria

**Figure 1:**
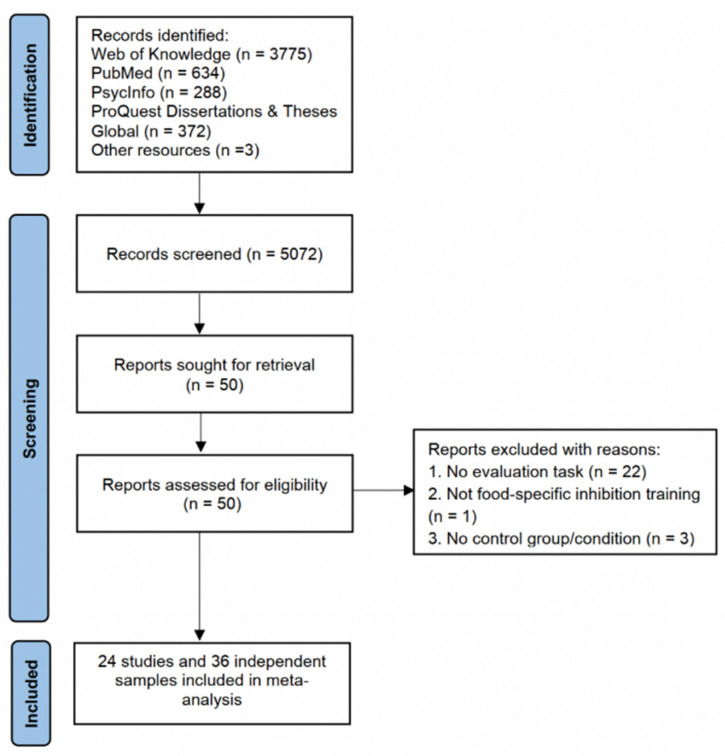
Flow diagram illustrating the process of our review, screening, and article selection processes.

The meta-analysis was performed according to the Preferred Reporting Items for Systematic Reviews and Meta-Analyses guidelines (see Supplementary Materials). A protocol for this work was registered on the Open Science Framework (OSF: https://osf.io/48p2r (accessed on 26 January 2022)). To obtain studies for use in the meta-analysis, we performed a comprehensive search of the databases PubMed, ISI Web of Knowledge, PsycINFO, and ProQuest Dissertations and Theses, using the search string presented in the Supplementary Materials. We concluded this search in January 2022. Abstracts of articles were reviewed, and the full text of an article was read whenever a paper’s title or abstract indicated that the study might be relevant to our analysis. In addition, to ensure that our review was comprehensive, the forward and backward citations of all eligible papers were searched manually for relevant studies. Furthermore, we conducted numerous non-exhaustive searches of Google Scholar using simple strings such as (“inhibition training” AND “food”) and similar variations. Figure 1 presents the flow diagram. 

Studies were incorporated into the meta-analysis if they (a) studied human participants, (b) focused on food-specific inhibition training, and (c) used at least one control group (between-subjects design) or condition (within-subjects design). It should be noted that if studies used a within-subjects design and only compared the no-go/stop food evaluation with the go food evaluation, we excluded these studies because, in such cases, it is unclear whether the food devaluation reflects an effect of the go food or an effect of the no-go/stop food [45] (for more information, please see Section 2.3). To warrant inclusion in the analysis, studies also had to (d) assess food evaluation and (e) provide data or statistical information that allowed for effect size calculation. If an article did not include sufficient information for effect size analysis, and the article was published within the last 10 years, the corresponding author was contacted, the reasoning being that older data were unlikely to be retained. The first author screened the full texts and extracted data from the selected studies, while the third author checked the data for accuracy.

### 2.2. Coding of Variables

Training tasks were coded according to their use of the go/no-go or stop-signal task as the training method.

Food novelty was coded as trained food if the evaluation task used the same food stimuli as those used in the training task, was coded as generalized food if the evaluation task used new food stimuli, and was coded as mixed food if the evaluation task used both original and new food stimuli.

The evaluation was coded as an implicit evaluation if the food evaluation task measured implicit evaluations, and was coded as an explicit evaluation if participants were shown food images or real food and asked to respond to questions according to a visual analogue scale (e.g., “How attractive does this food item look to you?”).

In samples of adult participants, a group was defined as being overweight or obese if the average body mass index (BMI) was recorded as 25 kg/m^2^ or above, or normal weight if the average BMI was between 18.5 and 24.99 kg/m^2^. In samples of children and adolescents, excessive weight or obesity was defined as a BMI at or above the 85th percentile, and normal weight as a BMI between the 5th and 84.99th percentiles. Five studies did not report the average BMI or the weight status of participants. However, because the participants of these studies were predominantly college or primary school students, we took the decision to code these participants as normal weight.

Length of follow-up was coded as immediate assessment if the studies assessed food evaluation immediately after training, and was coded as post-assessment if the studies assessed food evaluation with time delays (all other time frames).

### 2.3. Statistical Analysis

The effect size measurement used was the standardized mean difference between the active training and control groups. Hedges’ *g*—rather than Cohen’s *d*—was used as the effect size for analysis, given that the former is a relatively unbiased estimate of the standardized mean difference, while the latter is a biased estimate.

To calculate effect sizes for between-subjects designs, we used two different formulae. For post-test-only control group designs, we used the mean scores and the associated *SD*s for training and control groups in the post-test to calculate effect size [46]. For pre-test–post-test control group designs, mean scores and the associated *SD*s for training and control groups in the pre- and post-tests were used to calculate effect size [47]. The correlation between food evaluation in the pre-test with food evaluation in the post-test is needed for pre-test–post-test control group designs in order to calculate the variances. Fortunately, many of the studies considered in this meta-analysis used open-source statistics and shared their raw data on many websites, such as the Open Science Framework. Based on these shared data [14,34,48,49,50,51,52,53], we conducted a mini meta-analysis to calculate the “true” correlation, and the correlation was accordingly set at *r* = 0.78.

For within-subjects designs, we used *t* values from the paired sample *t*-test of post- minus pre-training evaluation change scores for no-go/stop and untrained foods to calculate the Cohen’s *d* value. To calculate variances, the correlation between food evaluation in the pre-test and food evaluation in the post-test are also needed. Similar to between-subjects designs, we also conducted a mini meta-analysis (including studies [26,30,35,40,54]) to calculate the “true” correlation between pre- and post-tests, and the correlation was accordingly set at *r* = 0.54. In addition, for within-subjects designs, we converted effect size estimates and their variances into the between-subjects effect size metric described by Morris and DeShon [55]. Lastly, we applied Hedges’ *g* correction function to all individual effect sizes [46].

It should be noted that many studies often feature more than one type of food evaluation (e.g., liking and attractiveness). Multiple outcomes are a problem for conventional meta-analytic methods, as averaging effect sizes within studies without accounting for their correlations can alter or obscure true effect size estimates [56]. Thus, we employed the meta-analytic technique of robust variance estimation—a random-effects meta-regression that can account for dependence between effect size estimates [57,58]. This technique gives a robust estimation of effect size weights and standard errors for the given effects, allowing for multiple outcomes within studies. We employed the robu() function of the robumeta package in R to conduct these analyses using the correlated weights given by Hedges, Tipton, and Johnson [57], with our primary analyses using the small sample corrections suggested by Tipton [59]. To account for dependency, ρ was set to 0.80 as recommended [58]. Heterogeneity was quantified as *τ*^2^, which represents between-study variance in this meta-analytic method [60].

Finally, we used the procedures described by Viechtbauer and Cheung [61] to derive extreme outliers (identified by inspecting the z-score of the standardized residuals) and influential studies (identified by inspecting Cook’s distance plots) (see Figure S1 for the plot of influence diagnostics). If the z-score of the standardized residuals exceeded 1.96, the study was deemed to be an outlier, and if Cook’s distance plots showed the outlier to exert a statistically significant influence on the results, the outlier was excluded, and only results from the meta-analysis without the outlier were reported in full.

For all of the following analyses, a positive effect size means that, relative to the control group, food-specific inhibition training decreased food evaluation, whereas a negative effect size indicates that, relative to the control group, food-specific inhibition training increased food evaluation. In addition, because the outcome in these analyses is the standardized mean difference between groups (the effect size), a statistically significant moderator means that the effect size estimate depends upon levels of that variable.

### 2.4. Quality Assessment

The 13-item quality scale for intervention studies developed by Thompson et al. [62] was used to assess the quality of the selected studies (see the Supplementary Materials for the quality of each included study).

## 3. Results

### 3.1. Preliminary Analysis

#### 3.1.1. Study Characteristics

Our search identified 24 eligible studies (total m = 24) [14,17,26,30,34,35,40,41,42,48,49,50,51,52,53,54,63,64,65,66,67,68,69,70], 36 independent samples (total k = 36), and a total of 3032 participants (total N = 3032). A complete list of studies and their characteristics can be seen in Table 1. From these studies, we obtained 77 effect sizes, which is similar to the number of effect sizes per study obtained in similar meta-analyses [43,71].

#### 3.1.2. Assessment of Publication Bias

**Figure 2:**
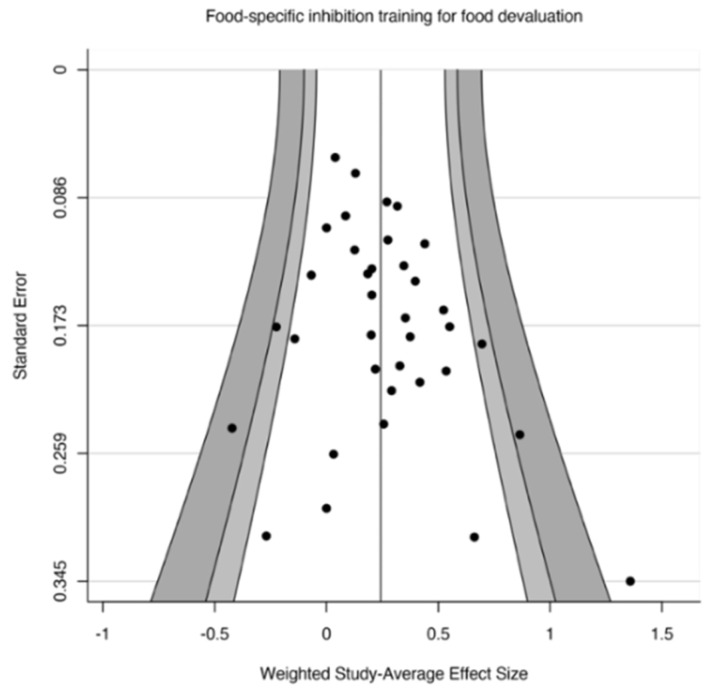
Funnel plot to ascertain evidence for publication bias in food-specific inhibition training on food evaluation.

To assess publication bias, we conducted Egger’s test [72] for funnel plot asymmetry in the effect of food-specific inhibition training on food evaluation (Figure 2). The results of Egger’s test were found to be nonsignificant (*t*(34) = 0.64, *p* = 0.525), indicating a lack of evidence for publication bias in these effects.

#### 3.1.3. Power Analysis

To ensure that our study had sufficient power to detect effects, we conducted power analysis for our random-effects meta-analysis. We used the average sample size for the training and control groups as the “typical” sample size per group, as well as the observed heterogeneity (*τ*^2^), to demonstrate the actual power of our analyses. Results showed that our analysis was extremely well powered (see Supplementary Materials, Table S2), with approximately 100% power to detect even small effects (e.g., |*g*^+^| = 0.20).

### 3.2. Overall Training Effect and Moderator Analyses

**Figure 3:**
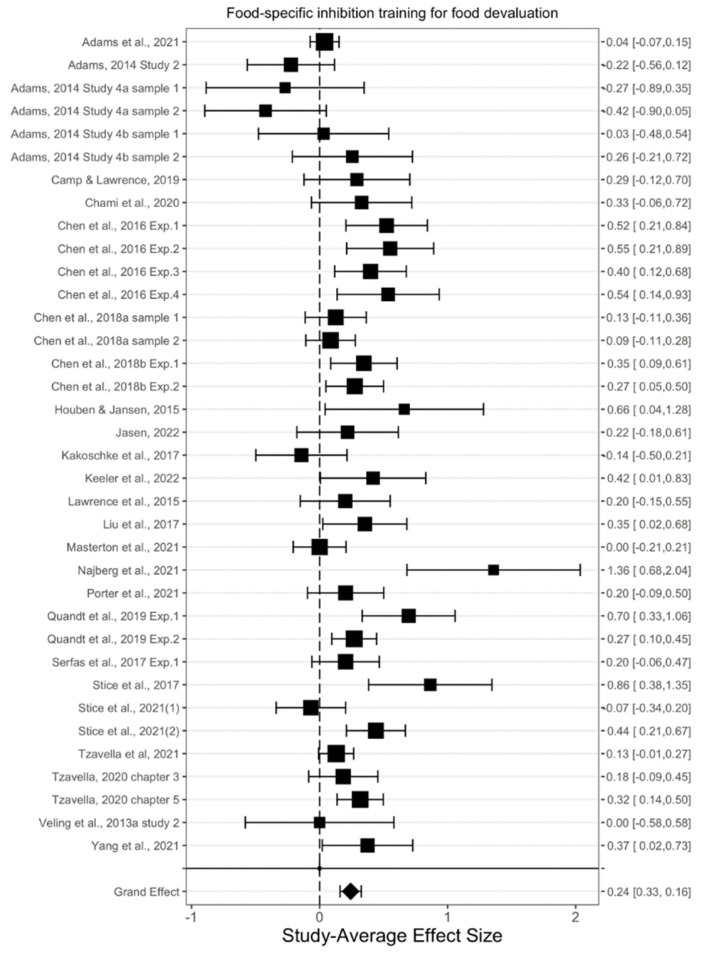
The effects of food-specific inhibition training on food evaluation [14,17,26,30,34,40,41,42,48,49,50,51,52,53,54,63,64,65,66,67,68,69,70].

The overall effect of food-specific inhibition training on food evaluation was found to be statistically significant (*g*^+^ = 0.242, *t*(31.6) = 5.97, *p* < 0.001, 95% CI_g_ [0.160, 0.325]) (see Figure 3), indicating that food-specific inhibition training decreased participants’ food evaluation. There was low heterogeneity observed across these effects (*τ*^2^ = 0.03), indicating that the effects of the training on food evaluation were relatively consistent across various conditions. Nevertheless, we explored the effects of moderators that were expected a priori to play an important role in the effects of food-specific inhibition training (Table 2).

We first examined whether the type of evaluation moderated the effects of the training on food evaluation, given that the evidence for the effect of food-specific inhibition training on implicit food evaluation is relatively weak. Results showed that the type of evaluation moderated the effects of training on food evaluation (*t*(5.55) = 3.23, *p* = 0.020); food-specific inhibition training significantly decreased participants’ explicit food evaluation (*g*^+^ = 0.285, *p* < 0.001), but not their implicit food evaluation (*g*^+^ = −0.100, *p* = 0.425).

We next examined whether the training model moderated the effects of the training on food evaluation, given prior work suggesting that the go/no-go task has a larger effect on changing unhealthy behaviors (e.g., eating behaviors) than the stop-signal task [e.g., 33]. Results showed that the training paradigm did not moderate the effects of food-specific inhibition training on food evaluation (*F*(1, 2.24) = 0.16, *p* = 0.728).

We then examined whether food novelty moderated the effects of the training on food evaluation. Results showed that food-specific inhibition training significantly lowered the evaluation of trained food (*g*^+^ = 0.291, *p* < 0.001), but not the evaluation of generalized food (*g*^+^ = 0.130, *p* = 0.108) or mixed food (*g*^+^ = 0.150, *p* = 0.271); however, these differences were not found to be statistically significant (*F* (1, 8.07) = 4.33, *p* = 0.071).

Furthermore, we examined whether weight status moderated the effects of food-specific inhibition training on food evaluation. Results showed that the effects (*g*^+^ = 0.328, *p* = 0.007) for overweight or obese individuals were not significantly different from the effects (*g*^+^ = 0.225, *p* < 0.001) for normal-weight individuals (*t*(8.35) = 1.24, *p* = 0.316).

Finally, exploratory analysis showed that none of age, sex, or length of follow-up moderated the effects of food-specific inhibition training on food evaluation (all *p* > 0.626).

## 4. Discussion

Many people today live in an obesogenic food environment, and are constantly exposed to low-nutritive-value yet appetitive foods—for example, foods containing large amounts of sugar and fat [73]. These environmental conditions paired with internal factors (e.g., impulsivity) could lead to higher food evaluation (e.g., food cravings), which might aggravate dysfunctional eating behaviors such as bulimia nervosa or binge eating [3]. Interventions aimed at lowering the evaluation of appetitive foods, therefore, may help people to regulate their eating behavior or body weight. In this review, we conducted the first—to our knowledge—systematic review and meta-analysis of studies examining the effects of food-specific inhibition training on food devaluation. We also explored the effects of several moderator variables that previous studies indicated might be critical for food devaluation. This comprehensive review of 36 independent samples—with 77 effect sizes and 3032 participants—revealed that, relative to the control condition, food-specific inhibition training significantly altered individuals’ food evaluation (*g*^+^ = 0.242, *p* < 0.001). Among seven moderators examined, we found that the effect of food-specific inhibition training was significantly moderated by the type of evaluation (*t*(5.55) = 3.23, *p* = 0.020). In particular, food-specific inhibition training significantly decreased explicit (*g*^+^ = 0.285, *p* < 0.001) but not implicit food evaluation (*g*^+^ = −0.100, *p* = 0.425). These results are discussed below, with a focus on the theoretical and practical applications.

There are various theories to explain the food devaluation effect of food-specific inhibition training. For example, the BSI theory [26,28] proposes that the conflict between the automatic approach tendency triggered by appetitive food stimuli and the task requirement of inhibition during the go/no-go or stop-signal task elicited a negative effect, which might be attached to specific food items and cause food devaluation. In addition, some researchers have argued that stopping and avoidance are linked to an aversive system [31,32], and this association might spill over to the responses to the no-go/stop food stimuli presented during training [34].

The food devaluation effect observed in the meta-analysis is supported by recent neuroimaging studies [14,67]. More specifically, researchers have found that, compared with changes observed in controls, food-specific inhibition training reduces activation in reward regions of the brain (e.g., putamen, mid-insula) in response to no-go/stop food images. Critically, activation change in the reward regions of the brain in response to the no-go/stop images was positively associated with changes in the evaluation of these images (e.g., *r* =0.44). Although such results are still nascent, these functional magnetic resonance imaging (*f*MRI) studies and future neuroscience studies similar to them can directly measure value signals in the brain, and may provide further conclusive evidence for the food devaluation effect of food-specific inhibition training [37].

The magnitude of the food devaluation effect observed in the meta-analysis was small-to-medium. Future studies should examine whether this small-to-medium effect size has any practical or real-world significance. For example, it was proposed that the decrease in food evaluation may play a critical role in promoting healthier eating behaviors. Supporting this notion are the findings of Veling et al. [74], who showed that the effect of food-specific inhibition training on food choices was entirely mediated by decreased evaluation of the foods that had been associated with the no-go cues. However, this study only assessed hypothetical choices and not actual, consequential behavior. Lawrence et al. [51] assessed self-reported eating behaviors and weight loss, but did not observe any evidence of mediation. Further (preregistration) studies are needed to investigate whether this food devaluation effect could act as a mechanism underlying the positive effects of food-specific inhibition training in terms of changing peoples’ eating behaviors.

### 4.1. Discussion of Moderators

To further highlight contextual factors that may influence the training effects, this meta-analysis examined the effects of potential moderators, including the type of evaluation, training paradigm, food novelty, weight status, age, sex, and length of follow-up.

Our results indicated that the effect of food-specific inhibition training on food evaluation was moderated by the type of evaluation. In particular, food-specific inhibition training was found to have a statistically significant effect on explicit food evaluation, but not on implicit food evaluation. Similarly, a previous meta-analysis showed that repeated inhibition of behaviors in response to appetitive stimuli (mainly alcohol stimuli) does not change implicit evaluation of these stimuli [75]. Taken together, current evidence suggests that stimulus-related inhibition training only changes explicit stimulus evaluation, which might provide further insights into how motor response training influences behavior. For example, it might be more likely that stimulus-related inhibition training changes behavior via changes in the explicit (but not the implicit) evaluation of stimuli, given the robust effect of training on the former.

We found no other statistically significant moderators. Researchers have speculated that, compared to the stop-signal task, higher food-inhibition contingency in the go/no-go task might result in a greater degree of effectiveness [76]. In this meta-analysis, both the go/no-go task and the stop-signal task showed high food-inhibition contingency (e.g., above 75%). However, we included only three studies that used the stop-signal task, which might have resulted in a lack of power to detect a moderation effect by the training paradigm.

Food novelty was also found not to moderate the effects of food-specific inhibition training on food evaluation. A series of experiments conducted by Chen et al. [26,40] showed a lack of generalization of training effects to untrained or novel food items when training was focused at the item level (e.g., similar food items appear on go and no-go trials). However, there has been evidence to suggest that food-specific inhibition training could be generalized to untrained stimuli when training is focused on a category level (e.g., healthy food = go; unhealthy food = no-go) [34,48,50]. Combining these studies, our meta-analysis showed that, although the effects of food-specific inhibition training on the evaluation of trained food (*g*^+^ = 0.291) were numerically larger than on the evaluation of generalized food (*g*^+^ = 0.130) or mixed food (*g*^+^ = 0.150), the differences were not statistically significant. It should be noted that there were only eight and nine studies that investigated the effects of training on generalized food and mixed food, respectively, which might result in an insufficiency of statistical power when conducting the moderator analysis. Therefore, future studies or meta-analyses should continue to test the generalization effects of food-specific inhibition training.

Weight status also did not emerge as a statistically significant moderator, suggesting that food-specific inhibition training causes similar decreases in food evaluation in normal-weight and overweight/obese participants. However, it should be noted that only seven food-specific inhibition training studies were conducted with people with excessive weight or obesity. Therefore, more studies focusing on overweight or obese individuals are needed before food-specific inhibition training can be translated into clinical interventions.

### 4.2. Limitations and Future Directions

Despite its strengths, this meta-analysis has limitations. Firstly, relative to the main analysis, statistical power might be low for some of the moderator analyses. For example, as previously mentioned, not many studies recruited people with excessive weight or obesity, or focused on whether there were generalization effects of food-specific inhibition training. Similarly, only four studies investigated the persistence of the training effects on food evaluation, and only three studies used the stop-signal task. As such, when additional related studies have been conducted, updated moderator analyses will be warranted. Secondly, there may be additional moderators (e.g., hunger, emotional or restrained eating, awareness) of the effects of food-specific training on food evaluation that were unaccounted for in our analysis. For example, Chen et al. [35] only observed the devaluation effect of food-specific inhibition training in relatively hungry participants. Adams et al. [34] suggested that some effects of food-specific inhibition training may be driven by awareness. However, most studies included in this meta-analysis failed to report these important aspects, precluding moderator analysis of these variables. Similarly, the large variability of types of explicit food evaluations also precludes moderator analysis of this variable. As such, we do not claim to present a complete picture of moderators. In summary, future studies should provide a full characterization of the participants and explore whether additional factors moderate the effects of food-specific inhibition training on food evaluation. Furthermore, the sole focus of our meta-analysis was food stimuli; future studies and meta-analyses could investigate whether food-specific inhibition training can also decrease the evaluations of other health-related stimuli, such as alcohol or cigarette cues. Finally, the preregistered protocol of current meta-analysis was minimal. We only preregistered the main aim and measured variables of the current study, and we recommend that future studies and meta-analyses carefully reflect on their study plans and include other important information (e.g., hypotheses and statistical analyses) in their preregistrations.

## 5. Conclusions

In conclusion, our meta-analysis supports the idea that food-specific inhibition training can produce beneficial changes in food evaluation. In particular, the type of evaluation moderated the effects of training on food evaluation, with food-specific inhibition training significantly decreasing participants’ explicit, but not their implicit, food evaluation. However, since most of the included studies focused on trained food items and short-term outcomes in normal-weight samples, more research is needed on the persistence of the training effects, and on the extent to which the effects can be generalized to untrained food items or different populations (e.g., overweight or obese individuals). 

## Figures and Tables

**Table 1:** Summary of the included papers for the effects of food-specific inhibition training on food evaluation.

Study	Participants	Training Condition	Control Condition	Session(s); Critical Trials	Study Design	Unhealthy Stimulus	Evaluation Type
Adams et al., 2021 [34]	N: 166/167 in training group; 146/141 in control group; Mean age: 23.69; Percent female: 77%; Inclusion criteria: N.A; Exclusion criteria: aged below 18 years or body mass index (BMI) < 18.5.	Inhibit 100 % of energy-dense food images	Filler images: 50% inhibit, 50% go	4/7; 216/378	Pre-test–post-test–control	Energy-dense food	Explicit: Liking taste
Adams, 2014 Study 2 [42]	N: 67 in training group; 65 in control group; Mean age: 23.12; Percent female: 93%; Inclusion criteria: chocolate cravers or restrained eaters; Exclusion criteria: currently dieting or any history of eating disorders.	Inhibit 87.5% of chocolate images	Filler images: 87.5% go, 12.5% inhibit	1; 70	Post-test only with control	Chocolate	Implicit: Implicit association test
Adams, 2014 study 4 sample 1 [42]	N: 13/38 in training group; 41/31 in control group; Mean age: 20.77/21.16; Percent female: 69%/95%; Inclusion criteria: N.A; Exclusion criteria: N.A.	Inhibit 100% of unhealthy snack foods	Filler images: 50% inhibit, 50% go	1; 36	Post-test only with control	Unhealthy snack foods	Implicit: Implicit association test
Adams, 2014 study 4 sample 2 [42]	N: 30/39 in training group; 28/31 in control group; Mean age: 24.47/21.41; Percent female: 67%/90%; Inclusion criteria: N.A; Exclusion criteria: N.A.	Inhibit 100% of unhealthy snack foods	Filler images: 50% inhibit, 50% go	1; 36	Post-test only with control	Unhealthy snack foods	Explicit: Attractiveness; Tastiness; Desire to Eat
Camp and Lawrence, 2019 [48]	N: 37 in training group; 30 in control group; Mean age: 24.1; Percent female: 85%; Inclusion criteria: 18–65 years, ate meat, and had some desire to reduce meat intake; Exclusion criteria: N.A.	Inhibit 100 % of meat	Filler images: 50% inhibit, 50% go	4; 192	Pre-test–post-test–control	Meat	Explicit: Liking
Chami et al., 2020 [49]	N: 28 in training group; 26 in control group; Mean age: 33.38; Percent female: 90%; Inclusion criteria: bulimia nervosa or binge-eating disorder, BMI > 18.5; Exclusion criteria: currently pregnant, had a visual impairment, a neurological impairment, alcohol or drug dependence, or psychosis.	Inhibit 100% of high-energy dense foods food	Filler images: 50% inhibit, 50% go	13.81; 756	Pre-test–post-test–control	High-energy dense foods food	Explicit: Liking
Chen et al., 2016 [26]	N: 41/38/43/27 in training group; Percent female: 83%/79%/89%; Mean age: 21.7/22.6/23.8/23.3; Inclusion criteria: N.A; Exclusion criteria: participants whose accuracy on go or no-go trials was 3 SD below sample mean and below 90%.	Inhibit 100% of palatable foods	Untrained	1; 50;100;60	Single-group Pre-test–post-test	Palatable foods	Explicit: Attractiveness
Chen et al., 2018a [35]	N: 59/58 in training group; Percent female: 76%/74%; Mean age: 46.1/23.2; Inclusion criteria: N.A; Exclusion criteria: N.A.	Inhibit 100% of palatable foods	Untrained	1; 108	Single-Group pre-test–post-test	Appetitive food	Explicit: Attractiveness
Chen et al., 2018b [40]	N: 71/106 in training group; Percent female: 89%/72%; Mean age: 20.7/23.2; Inclusion criteria: N.A; Exclusion criteria: N.A.	Inhibit 100% of palatable foods	Untrained	1; 30	Single-Group pre-test–post-test	Palatable foods	Explicit: Attractiveness
Houben and Jansen, 2015 [17]	N: 21 in training group; 20 in control group; Mean age: 20.1; Percent female: 100%; Inclusion criteria: liked to eat chocolate on a regular basis; Exclusion criteria: had severe to moderate underweight (BMI < 18.5), disliked the chocolate that was presented during the taste test (mean rating < 5), or were outliers.	Inhibit 100 % of chocolate snacks	Inhibit 0% chocolate snacks	1; 80	Post-test only with control	Chocolate snacks	Explicit: Craving
Jansen, 2022 [63]	N: 19/22 in training group; 23 in control group; Mean age: 44.8; Percent female: 83%; Inclusion criteria: aged 18 or older, a BMI ≥ 25, having a desire to lose weight, and consuming at least one of the no-go training foods used in the training at least two times per week; Exclusion criteria: medical condition limiting dietary intake or affecting weight, use of weight loss medication, history of bariatric surgery, current smoker, having quit smoking within the past year, or enrollment in a formal weight loss program in the past 6 months.	Inhibit 100 % of unhealthy foods	Inhibit 0% unhealthy foods	16/4; 864/216	Pre-test–post-test–control	Unhealthy foods	Explicit: Tastiness
Kakoschke et al., 2017 [41]	N: 60 in training group; 60 in control group; Mean age: 20.6; Percent female: 100%; Inclusion criteria: liked most foods, and did not have any food allergies, intolerances, or special dietary requirements; Exclusion criteria: N.A.	Inhibit 90% of unhealthy food	Inhibit 0% unhealthy food	1; 144	Post-test only with control	Unhealthy food	Implicit: Implicit association test
Keeler et al., 2022 [50]	N: 40 in training group; 40 in control group; Mean age: 30; Percent female: 98%; Inclusion criteria: bulimia nervosa or binge-eating disorder, receiving a form of treatment for their eating disorder (one or more of: psychotherapies, nutritional support, and/or psychiatric medications such as anti-depressants), had a BMI of at least 18.5 kg/m^2^, were between the ages of 18 and 60; Exclusion criteria: currently pregnant, had a visual impairment that could not be repaired with eyewear, a neurological impairment, alcohol or drug dependence, or psychosis.	Inhibit 100% of high energy-dense food and treatment-as-usual	Treatment-as-usual	21; 168	Pre-test–post-test–control	High energy-dense food	Explicit: Attractiveness
Lawrence et al., 2015a [51]	N: 42 in training group; 42 in control group; Mean age: 50; Percent female: 76%; Inclusion criteria: aged 18–65, had a BMI based on self-reported height and weight of at least 18.5, consumed some of the “no-go” snack foods (see below) at least three times per week, and reported some disinhibition over eating; Exclusion criteria: allergies to the foods given during the taste test, smoking/recent smoking cessation, enrolment in a formal weight loss program, use of weight loss medication, metabolic disorders, or other health conditions affecting weight.	Inhibit 100% of energy-dense food	Filter images: 50% inhibit, 50% go	4; 216	Pre-test–post-test–control	Energy-dense food	Explicit: Attractiveness; Liking
Liu et al., 2017 [64]	N: 33 in training group; 33 in control group; Mean age: 50; Percent female: 76%; Inclusion criteria: BMI between 18.5–23.9, restrained eater; Exclusion criteria: N.A.	Inhibit 87.5% of high-energy density foods	Filter images: 87.5% inhibit, 12.5% go	7; 588	Pre-test–post-test–control	High-energy density foods	Explicit: Attractiveness; Liking; Implicit: Implicit association test
Masterton et al., 2021 [52]	N: 47/44 in training group; 35/44 in control group; Mean age: 28.5/28.0; Percent female: 57%/50%; Inclusion criteria: N.A; Exclusion criteria: N.A.	Inhibit 100%/75% of unhealthy food	Inhibit 25%/50% of unhealthy food images	1; 100/75	Pre-test–post-test–control	Unhealthy food	Explicit: Appealing
Najberg et al., 2021 [53]	N: 46 in training group; 44 in control group; Mean age: 25.2; Percent female: 59%; Inclusion criteria: healthy individuals, BMI > 20, liking of unhealthy food; Exclusion criteria: consumption of any prescribed medication, diagnosis of eating disorders, restrictive diet, history of weight gain/loss of more than 10% body weight in the last six months, no plan of actively losing weight with a restrictive diet in the next four months.	Inhibit 100% unhealthy food	Inhibit 50% of unhealthy food images	20; n.a	Pre-test–post-test–control	Unhealthy food	Explicit: Palatability
Porter et al., 2021 [65]	N: 67/69 in training group; 64 in control group; Mean age: 7/6.6; Percent female: 53%/44%; Inclusion criteria: N.A; Exclusion criteria: N.A.	Inhibit 100% of energy-dense food	Filter images: 50% inhibit, 50% go	1; 96/80	Pre-test–post-test–control	Energy-dense food	Explicit: Yummy
Quandt et al., 2019 [30]	N: 41/79 in training group; Mean age: 22.6/22.4; Percent female: 78%; Inclusion criteria: N.A; Exclusion criteria: correct at least 90% of the time during training.	Inhibit 100% of palatable food	Untrained	1; 100	Single-group pre-test–post-test	Palatable food	Explicit: Appealing
Serfas et al., 2017 [66]	N: 51 in training group; Mean age: 26.7; Percent female: 47%; Inclusion criteria: N.A; Exclusion criteria: N.A.	Inhibit 100% of attractive food	Untrained	1; 40/50	Single-group pre-test–post-test	Attractive food	Explicit: Attractiveness
Stice et al., 2017 [67]	N: 21 in training group; 26 in the control group; Mean age: 19.2; Percent female: 95%; Inclusion criteria: weight concerns and a BMI of 25 or greater Exclusion criteria: current DSM-IV anorexia nervosa, bulimia nervosa, or binge-eating disorder.	Inhibit 100% of high-calorie foods	Inhibit 0% of high-calorie foods	4; 1120	Pre-test–post-test–control	High-calorie foods	Explicit: Palatability and monetary value
Stice et al., 2021 [68]	N: 21 in training group; 26 in the control group; Mean age: 19.2; Percent female: 95%; Inclusion criteria: between 17 and 20 years of age, had a BMI greater than 20 and less than 30, and reported concern about their weight; Exclusion criteria: a current diagnosis of anorexia nervosa, bulimia nervosa, or binge-eating disorder.	Inhibit 100% of high-calorie foods	Inhibit 0% of high-calorie foods	6; 840	Pre-test–post-test–control	High-calorie foods	Explicit: Palatability and monetary value
Tzavella et al., 2021 [54]	N: 163 in training group; Mean age: 22.4; Percent female: 81%; Inclusion criteria: at least 18 years of age, fluent in spoken and written English, and normal or corrected-to-normal vision; Exclusion criteria: dieting at the time of the study, with a weight goal and timeframe in mind, current and/or past diagnosis of any eating disorder(s), or a BMI lower than 18.5 kg/m^2^.	Inhibit 100% of energy-dense foods	Untrained	1; 72	Single-group pretest–post-test	Energy-dense foods	Explicit: Liking
Tzavella et al., 2020 [69]	N: 96/117/113 in training group; Mean age: 21.6/26.9; Percent female: 57%; Inclusion criteria: at least 18 years of age, with normal or corrected-to-normal vision; Exclusion criteria: not able to understand written and spoken English well, reported having a food allergy and/or intolerance to any of the major food allergens, or had a self-reported past or current diagnosis of an eating disorder, with the exception of binge-eating disorder.	Inhibit 100% of energy-dense foods	Untrained	1; 64/128	Post-test only with control/Single-group pre-test–post-test	Energy-dense foods	Explicit: Liking; craving Implicit: Affective priming paradigm
Veling et al., 2013a study 2 [70]	N: 22 in training group; 22 in the control group; Mean age: 21.5; Percent female: 61%; Inclusion criteria: N.A; Exclusion criteria: N.A.	Inhibit 100% of snack foods	Snack foods: 0% inhibit	1; 32	Post-test only with control	Snack foods	Explicit: Palatability
Yang et al., 2021a [14]	N: 21 in training group; 26 in the control group; Mean age: 19.2; Percent female: 95%; Inclusion criteria: had weight concerns, were willing to participate in the current weight control trials, and had a BMI of 23 or greater; Exclusion criteria: self-reported current eating disorders, mental disorders, or head injuries.	Inhibit 100% of energy-dense foods	Filter image: 50% go, 50 inhibit	5; 500	Pre-test–post-test–control	Energy-dense foods	Explicit: Attractiveness

Note: N.A = not available.

**Table 2:** Moderator analysis of the effects of food-specific inhibition training on food evaluation.

Moderator	*β*	*t*/*F* (df)	k	*g* ^+^	*p*
Participant age	0.001	0.22 (4.5)			0.834
Percentage of female participants	0.089	0.30 (10.1)			0.771
Type of evaluation		3.23 (5.57)			**0.020**
Explicit evaluation			30	0.285	<0.001
Implicit evaluation			6	−0.100	0.425
Training paradigm		0.16 (2.24)			0.728
Go/no-go task			30	0.247	<0.001
Stop-signal task			3	0.112	0.556
Mixed			3	0.341	0.296
Food novelty		4.33 (8.07)			0.071
Trained food			19	0.291	<0.001
Generalized food			8	0.130	0.108
Mixed			9	0.150	0.271
Weight status		1.24 (8.35)			0.316
Normal weight			29	0.225	<0.001
Overweight/obesity			7	0.328	0.007
Length of follow-up		−0.57 (2.02)			0.626
Immediate			32	0.246	<0.001
Post			4	0.193	0.189

Note: Significant (*p* < 0.05) moderating effects are listed in bold font.

## Data Availability

Datasets arising from the study are available on OSF (https://osf.io/32gby/ (accessed on 26 January 2022)).

## Supplementary Materials

The following supporting information can be downloaded at: https://www.mdpi.com/article/10.3390/nu14071363/s1, Figure S1: Plot of Influence Diagnostics; Table S1: PRISMA 2020 Checklist; Table S2: Quality assessments for included studies; Table S3: Power Analyses Describing Achieved Power to Detect Effects of Food-specific Inhibition Training on Food Evaluation in a Two-Tailed Test.

## References

[1] De Graaf C., Blom W.A., Smeets P.A., Stafleu A., Hendriks H.F. (2004). Biomarkers of satiation and satiety. Am. J. Clin. Nutr..

[2] Yang Y., Wu Q., Morys F. (2021). Brain Responses to High-Calorie Visual Food Cues in Individuals with Normal-Weight or Obesity: An Activation Likelihood Estimation Meta-Analysis. Brain Sci..

[3] Oliveira J., Cordas T.A. (2020). The body asks and the mind judges: Food cravings in eating disorders. Encephale.

[4] Berridge K.C., Ho C.Y., Richard J.M., DiFeliceantonio A.G. (2010). The tempted brain eats: Pleasure and desire circuits in obesity and eating disorders. Brain Res..

[5] Stice E., Burger K. (2019). Neural vulnerability factors for obesity. Clin. Psychol. Rev..

[6] Stice E., Yokum S. (2016). Neural vulnerability factors that increase risk for future weight gain. Psychol. Bull..

[7] Stice E., Lawrence N.S., Kemps E., Veling H. (2016). Training motor responses to food: A novel treatment for obesity targeting implicit processes. Clin. Psychol. Rev..

[8] Veling H., Lawrence N.S., Chen Z., van Koningsbruggen G.M., Holland R.W. (2017). What Is Trained During Food Go/No-Go Training? A Review Focusing on Mechanisms and a Research Agenda. Curr. Addict. Rep..

[9] Logan G.D., Cowan W.B., Davis K.A. (1984). On the ability to inhibit simple and choice reaction time responses: A model and a method. J. Exp. Psychol. Hum. Percept. Perform..

[10] Diamond A. (2013). Executive functions. Annu. Rev. Psychol..

[11] Shields G.S., Moons W.G., Slavich G.M. (2017). Inflammation, Self-Regulation, and Health: An Immunologic Model of Self-Regulatory Failure. Perspect. Psychol. Sci..

[12] Yang Y., Shields G.S., Wu Q., Liu Y., Chen H., Guo C. (2019). Cognitive training on eating behaviour and weight loss: A meta-analysis and systematic review. Obes. Rev..

[13] Forcano L., Mata F., de la Torre R., Verdejo-Garcia A. (2018). Cognitive and neuromodulation strategies for unhealthy eating and obesity: Systematic review and discussion of neurocognitive mechanisms. Neurosci. Biobehav. Rev..

[14] Yang Y., Morys F., Wu Q., Li J., Chen H. (2021). Pilot Study of Food-Specific Go/No-Go Training for Overweight Individuals: Brain Imaging Data Suggest Inhibition Shapes Food Evaluation. Soc. Cogn. Affect. Neurosci..

[15] Lawrence N.S., Verbruggen F., Morrison S., Adams R.C., Chambers C.D. (2015). Stopping to food can reduce intake. Effects of stimulus-specificity and individual differences in dietary restraint. Appetite.

[16] Guerrieri R., Nederkoorn C., Jansen A. (2012). Disinhibition is easier learned than inhibition. The effects of (dis)inhibition training on food intake. Appetite.

[17] Houben K., Jansen A. (2015). Chocolate equals stop. Chocolate-specific inhibition training reduces chocolate intake and go associations with chocolate. Appetite.

[18] Chen Z., Holland R.W., Quandt J., Dijksterhuis A., Veling H. (2019). When mere action versus inaction leads to robust preference change. J. Pers. Soc. Psychol..

[19] Porter L., Bailey-Jones C., Priudokaite G., Allen S., Wood K., Stiles K., Parvin O., Javaid M., Verbruggen F., Lawrence N.S. (2018). From cookies to carrots; the effect of inhibitory control training on children’s snack selections. Appetite.

[20] Veling H., Verpaalen I.A.M., Liu H., Mosannenzadeh F., Becker D., Holland R.W. (2021). How can food choice best be trained? Approach-avoidance versus go/no-go training. Appetite.

[21] Wolz I., Nannt J., Svaldi J. (2020). Laboratory-based interventions targeting food craving: A systematic review and meta-analysis. Obes. Rev..

[22] Allom V., Mullan B., Hagger M. (2016). Does inhibitory control training improve health behaviour? A meta-analysis. Health Psychol. Rev..

[23] Veling H., Chen Z., Liu H., Quandt J., Holland R.W. (2020). Updating the p-curve analysis of Carbine and Larson with results from preregistered experiments. Health Psychol. Rev..

[24] Carbine K.A., Larson M.J. (2019). Quantifying the presence of evidential value and selective reporting in food-related inhibitory control training: A p-curve analysis. Health Psychol. Rev..

[25] Navas J.F., Verdejo-Garcia A., Vadillo M.A. (2021). The evidential value of research on cognitive training to change food-related biases and unhealthy eating behavior: A systematic review and p-curve analysis. Obes. Rev..

[26] Chen Z., Veling H., Dijksterhuis A., Holland R.W. (2016). How does not responding to appetitive stimuli cause devaluation: Evaluative conditioning or response inhibition?. J. Exp. Psychol. Gen..

[27] Johannes N., Buijzen M., Veling H. (2021). Beyond inhibitory control training: Inactions and actions influence smartphone app use through changes in explicit liking. J. Exp. Psychol. Gen..

[28] Veling H., Holland R.W., van Knippenberg A. (2008). When approach motivation and behavioral inhibition collide: Behavior regulation through stimulus devaluation. J. Exp. Soc. Psychol..

[29] Dreisbach G., Fischer R. (2015). Conflicts as Aversive Signals for Control Adaptation. Curr. Dir. Psychol. Sci..

[30] Quandt J., Holland R.W., Chen Z., Veling H. (2019). The role of attention in explaining the no-go devaluation effect: Effects on appetitive food items. J. Exp. Psychol. Hum. Percept. Perform..

[31] Guitart-Masip M., Duzel E., Dolan R., Dayan P. (2014). Action versus valence in decision making. Trends Cogn. Sci..

[32] Guitart-Masip M., Huys Q.J., Fuentemilla L., Dayan P., Duzel E., Dolan R.J. (2012). Go and no-go learning in reward and punishment: Interactions between affect and effect. Neuroimage.

[33] Verbruggen F., Best M., Bowditch W.A., Stevens T., McLaren I.P. (2014). The inhibitory control reflex. Neuropsychologia.

[34] Adams R.C., Button K.S., Hickey L., Morrison S., Smith A., Bolus W., Coombs E., Randolph S., Hunt R., Kim D. (2021). Food-related inhibitory control training reduces food liking but not snacking frequency or weight in a large healthy adult sample. Appetite.

[35] Chen Z., Veling H., de Vries S.P., Bijvank B.O., Janssen I.M.C., Dijksterhuis A., Holland R.W. (2018). Go/no-go training changes food evaluation in both morbidly obese and normal-weight individuals. J. Consult. Clin. Psychol..

[36] Wessel J.R., Aron A.R. (2017). On the Globality of Motor Suppression: Unexpected Events and Their Influence on Behavior and Cognition. Neuron.

[37] Wessel J.R., O’Doherty J.P., Berkebile M.M., Linderman D., Aron A.R. (2014). Stimulus devaluation induced by stopping action. J. Exp. Psychol. Gen..

[38] Chiu Y.C., Egner T. (2015). Inhibition-Induced Forgetting Results from Resource Competition between Response Inhibition and Memory Encoding Processes. J. Neurosci..

[39] Chiu Y.C., Egner T. (2015). Inhibition-induced forgetting: When more control leads to less memory. Psychol. Sci..

[40] Chen Z., Veling H., Dijksterhuis A., Holland R.W. (2018). Do impulsive individuals benefit more from food go/no-go training? Testing the role of inhibition capacity in the no-go devaluation effect. Appetite.

[41] Kakoschke N., Kemps E., Tiggemann M. (2017). The effect of combined avoidance and control training on implicit food evaluation and choice. J. Behav. Ther. Exp. Psychiatry.

[42] Adams R. (2014). Training Response Inhibition to Reduce Food Consumption. Doctoral Thesis.

[43] Yang Y., Shields G.S., Guo C., Liu Y. (2018). Executive function performance in obesity and overweight individuals: A meta-analysis and review. Neurosci. Biobehav. Rev..

[44] Stoeckel L.E., Weller R.E., Cook E.W., Twieg D.B., Knowlton R.C., Cox J.E. (2008). Widespread reward-system activation in obese women in response to pictures of high-calorie foods. Neuroimage.

[45] Schonberg T., Bakkour A., Hover A.M., Mumford J.A., Nagar L., Perez J., Poldrack R.A. (2014). Changing value through cued approach: An automatic mechanism of behavior change. Nat. Neurosci..

[46] Borenstein M., Hedges L.V., Higgins J.P., Rothstein H.R. (2009). Introduction to Meta-Analysis.

[47] Morris S.B. (2008). Estimating effect sizes from pretest-posttest-control group designs. Organ. Res. Methods.

[48] Camp B., Lawrence N.S. (2019). Giving pork the chop: Response inhibition training to reduce meat intake. Appetite.

[49] Chami R., Cardi V., Lawrence N., MacDonald P., Rowlands K., Hodsoll J., Treasure J. (2020). Targeting binge eating in bulimia nervosa and binge eating disorder using inhibitory control training and implementation intentions: A feasibility trial. Psychol. Med..

[50] Keeler J.L., Chami R., Cardi V., Hodsoll J., Bonin E., MacDonald P., Treasure J., Lawrence N. (2022). App-based food-specific inhibitory control training as an adjunct to treatment as usual in binge-type eating disorders: A feasibility trial. Appetite.

[51] Lawrence N.S., O’Sullivan J., Parslow D., Javaid M., Adams R.C., Chambers C.D., Kos K., Verbruggen F. (2015). Training response inhibition to food is associated with weight loss and reduced energy intake. Appetite.

[52] Masterton S., Hardman C.A., Halford J.C.G., Jones A. (2021). Examining cognitive bias modification interventions for reducing food value and choice: Two pre-registered, online studies. Appetite.

[53] Najberg H., Rigamonti M., Mouthon M., Spierer L. (2021). Modifying food items valuation and weight with gamified executive control training. R. Soc. Open Sci..

[54] Tzavella L., Lawrence N.S., Button K.S., Hart E.A., Holmes N.M., Houghton K., Badkar N., Macey E., Braggins A.J., Murray F.C. (2021). Effects of go/no-go training on food-related action tendencies, liking and choice. R. Soc. Open Sci..

[55] Morris S.B., DeShon R.P. (2002). Combining effect size estimates in meta-analysis with repeated measures and independent-groups designs. Psychol. Methods.

[56] Scammacca N., Roberts G., Stuebing K.K. (2014). Meta-Analysis With Complex Research Designs: Dealing With Dependence From Multiple Measures and Multiple Group Comparisons. Rev. Educ. Res..

[57] Hedges L.V., Tipton E., Johnson M.C. (2010). Robust variance estimation in meta-regression with dependent effect size estimates. Res. Synth. Methods.

[58] Tanner-Smith E.E., Tipton E. (2014). Robust variance estimation with dependent effect sizes: Practical considerations including a software tutorial in Stata and spss. Res. Synth. Methods.

[59] Tipton E. (2015). Small sample adjustments for robust variance estimation with meta-regression. Psychol. Methods.

[60] Borenstein M., Higgins J.P., Hedges L.V., Rothstein H.R. (2017). Basics of meta-analysis: I_2_ is not an absolute measure of heterogeneity. Res. Synth. Methods.

[61] Viechtbauer W., Cheung M.W. (2010). Outlier and influence diagnostics for meta-analysis. Res. Synth. Methods.

[62] Thompson T., Oram C., Correll C.U., Tsermentseli S., Stubbs B. (2017). Analgesic Effects of Alcohol: A Systematic Review and Meta-Analysis of Controlled Experimental Studies in Healthy Participants. J. Pain.

[63] Jansen E.T. (2022). The Effect of Go/No-Go Training Dosage on Weight Loss, Food Evaluation, and Disinhibition in Primarily Overweight and Obese Individuals: A Randomized Controlled Trial. Master’s Thesis.

[64] Liu Y., Chen H., Li S., Luo N. (2017). Reducing unsuccessful restrained eaters’ unhealthy food choice: An internet-based inhibition control training. Acta Psychol. Sin..

[65] Porter L., Gillison F.B., Wright K.A., Verbruggen F., Lawrence N.S. (2021). Exploring Strategies to Optimise the Impact of Food-Specific Inhibition Training on Children’s Food Choices. Front. Psychol..

[66] Serfas B.G., Florack A., Büttner O.B., Voegeding T. (2017). What does it take for sour grapes to remain sour? Persistent effects of behavioral inhibition in go/no-go tasks on the evaluation of appetitive stimuli. Motiv. Sci..

[67] Stice E., Yokum S., Veling H., Kemps E., Lawrence N.S. (2017). Pilot test of a novel food response and attention training treatment for obesity: Brain imaging data suggest actions shape valuation. Behav. Res. Ther..

[68] Stice E., Rohde P., Gau J.M., Butryn M.L., Shaw H., Cloud K., D’Adamo L. (2021). Enhancing efficacy of a dissonance-based obesity and eating disorder prevention program: Experimental therapeutics. J. Consult. Clin. Psychol..

[69] Tzavella L. (2020). Behavioural Measures and Training Interventions for Food-Related Cognition, Motivation and Affect. Ph.D. Thesis.

[70] Veling H., Aarts H., Stroebe W. (2013). Using stop signals to reduce impulsive choices for palatable unhealthy foods. Br. J. Health Psychol..

[71] Shields G.S., Bonner J.C., Moons W.G. (2015). Does cortisol influence core executive functions? A meta-analysis of acute cortisol administration effects on working memory, inhibition, and set-shifting. Psychoneuroendocrinology.

[72] Egger M., Davey Smith G., Schneider M., Minder C. (1997). Bias in meta-analysis detected by a simple, graphical test. BMJ.

[73] Swinburn B.A., Sacks G., Hall K.D., McPherson K., Finegood D.T., Moodie M.L., Gortmaker S.L. (2011). The global obesity pandemic: Shaped by global drivers and local environments. Lancet.

[74] Veling H., Aarts H., Stroebe W. (2013). Stop signals decrease choices for palatable foods through decreased food evaluation. Front. Psychol..

[75] Jones A., Di Lemma L.C., Robinson E., Christiansen P., Nolan S., Tudur-Smith C., Field M. (2016). Inhibitory control training for appetitive behaviour change: A meta-analytic investigation of mechanisms of action and moderators of effectiveness. Appetite.

[76] Jones A., Hardman C.A., Lawrence N., Field M. (2018). Cognitive training as a potential treatment for overweight and obesity: A critical review of the evidence. Appetite.

