# Supplementary material for: Food-Specific Inhibition Training for Food Devaluation: A Meta-Analysis
Source: Nutrients. 2022 Mar 24;14(7):1363. doi: 10.3390/nu14071363 (PMC9002952; doi:10.3390/nu14071363)
Supplement: Supplementary file 1 [file nutrients-14-01363-s001.zip › nutrients-1639891-supplementary.pdf]

Supplementary material for

Food-specific Inhibition Training for Food Devaluation: A Meta-analysis

1. Table S1 PRISMA 2020 Checklist

2. Table S2 Quality assessments for included studies

3. Search terms used for the meta-analysis

4. Figure S1 Plot of Influence Diagnostics

5. Table S3 Power Analyses Describing Achieved Power to Detect Effects of Food-specific Inhibition Training on Food Evaluation in a Two-Tailed Test.

**Table S1. PRISMA 2020 Checklist**			
**Section and Topic**	**Item #**	**Checklist item**	**Location where item is reported**
**TITLE**			
Title	1	Identify the report as a systematic review.	Page 1
**ABSTRACT**			
Abstract	2	See the PRISMA 2020 for Abstracts checklist.	Page 2
**INTRODUCTION**			
Rationale	3	Describe the rationale for the review in the context of existing knowledge.	Page 6
Objectives	4	Provide an explicit statement of the objective(s) or question(s) the review addresses.	Page 7
**METHODS**			
Eligibility criteria	5	Specify the inclusion and exclusion criteria for the review and how studies were grouped for the syntheses.	Page 8
Information sources	6	Specify all databases, registers, websites, organisations, reference lists and other sources searched or consulted to identify studies. Specify the date when each source was last searched or consulted.	Page 7

Search strategy	7	Present the full search strategies for all databases, registers and websites, including any filters and limits used.	Supplementary Material
Selection process	8	Specify the methods used to decide whether a study met the inclusion criteria of the review, including how many reviewers screened each record and each report retrieved, whether they worked independently, and if applicable, details of automation tools used in the process.	Page 8
Data collection process	9	Specify the methods used to collect data from reports, including how many reviewers collected data from each report, whether they worked independently, any processes for obtaining or confirming data from study investigators, and if applicable, details of automation tools used in the process.	Page 8
Data items	10a	List and define all outcomes for which data were sought. Specify whether all results that were compatible with each outcome domain in each study were sought (e.g. for all measures, time points, analyses), and if not, the methods used to decide which results to collect.	Page 8, 9
	10b	List and define all other variables for which data were sought (e.g. participant and intervention characteristics, funding sources). Describe any assumptions made about any missing or unclear information.	Table 1
Study risk of bias assessment	11	Specify the methods used to assess risk of bias in the included studies, including details of the tool(s) used, how many reviewers assessed each study and whether they worked independently, and if applicable, details of automation tools used in the process.	Page 11
Effect measures	12	Specify for each outcome the effect measure(s) (e.g. risk ratio, mean difference) used in the synthesis or presentation of results.	Page 9

Synthesis methods	13a	Describe the processes used to decide which studies were eligible for each synthesis (e.g. tabulating the study intervention characteristics and comparing against the planned groups for each synthesis (item #5)).	Page 8
	13b	Describe any methods required to prepare the data for presentation or synthesis, such as handling of missing summary statistics, or data conversions.	Page 10
	13c	Describe any methods used to tabulate or visually display results of individual studies and syntheses.	Page 10
	13d	Describe any methods used to synthesize results and provide a rationale for the choice(s). If meta-analysis was performed, describe the model(s), method(s) to identify the presence and extent of statistical heterogeneity, and software package(s) used.	Page 10
	13e	Describe any methods used to explore possible causes of heterogeneity among study results (e.g. subgroup analysis, meta-regression).	Page 11
	13f	Describe any sensitivity analyses conducted to assess robustness of the synthesized results.	Page 11
Reporting bias assessment	14	Describe any methods used to assess risk of bias due to missing results in a synthesis (arising from reporting biases).	Page 8
Certainty assessment	15	Describe any methods used to assess certainty (or confidence) in the body of evidence for an outcome.	Page 10
**RESULTS**			
Study selection	16a	Describe the results of the search and selection process, from the number of records identified in the search to the number of studies included in the review, ideally using a flow diagram.	Figure 1

	16b	Cite studies that might appear to meet the inclusion criteria, but which were excluded, and explain why they were excluded.	Figure 1
Study characteristics	17	Cite each included study and present its characteristics.	Table 1
Risk of bias in studies	18	Present assessments of risk of bias for each included study.	Supplementary Material
Results of individual studies	19	For all outcomes, present, for each study: (a) summary statistics for each group (where appropriate) and (b) an effect estimate and its precision (e.g. confidence/credible interval), ideally using structured tables or plots.	Figure 2
Results of syntheses	20a	For each synthesis, briefly summarise the characteristics and risk of bias among contributing studies.	Table 1
	20b	Present results of all statistical syntheses conducted. If meta-analysis was done, present for each the summary estimate and its precision (e.g. confidence/credible interval) and measures of statistical heterogeneity. If comparing groups, describe the direction of the effect.	Page 12
	20c	Present results of all investigations of possible causes of heterogeneity among study results.	Page 12
	20d	Present results of all sensitivity analyses conducted to assess the robustness of the synthesized results.	Supplementary Material
Reporting biases	21	Present assessments of risk of bias due to missing results (arising from reporting biases) for each synthesis assessed.	Page 12

Certainty of evidence	22	Present assessments of certainty (or confidence) in the body of evidence for each outcome assessed.	Page 12
**DISCUSSION**			
Discussion	23a	Provide a general interpretation of the results in the context of other evidence.	Page 14
	23b	Discuss any limitations of the evidence included in the review.	Page 17
	23c	Discuss any limitations of the review processes used.	Page 17
	23d	Discuss implications of the results for practice, policy, and future research.	Page 17
**OTHER INFORMATION**			
Registration and protocol	24a	Provide registration information for the review, including register name and registration number, or state that the review was not registered.	Page 7
	24b	Indicate where the review protocol can be accessed, or state that a protocol was not prepared.	Page 7
	24c	Describe and explain any amendments to information provided at registration or in the protocol.	Page 7
Support	25	Describe sources of financial or non-financial support for the review, and the role of the funders or sponsors in the review.	Page 1
Competing interests	26	Declare any competing interests of review authors.	Page 1

Availability of data, code and other materials	27	Report which of the following are publicly available and where they can be found: template data collection forms; data extracted from included studies; data used for all analyses; analytic code; any other materials used in the review.	Page 1

Table S2. Quality assessments for included studies

	Items													
**Study**	**1**	**2**	**3**	**4**	**5**	**6**	**7**	**8**	**9**	**10**	**11**	**12**	**13**	**Total**
Adams et al., 2021	1	N/A	1	1	0	1	1	1	1	1	0	0	1	9
Adams, 2014 Study 2	0	N/A	1	1	1	1	1	1	1	1	0	0	1	9
Adams, 2014 Study 4	1	N/A	1	1	0	1	1	1	1	1	0	0	1	9
Camp & Lawrence, 2019	1	0	1	1	0	1	1	1	1	1	1	0	1	10
Chami et al., 2020	1	0	1	1	1	1	1	1	1	1	0	0	1	10
Chen et al., 2016	1	N/A	1	1	0	1	1	1	1	1	N/A	N/A	1	9
Chen et al., 2018a	1	N/A	1	1	0	1	1	1	1	1	N/A	N/A	1	9
Chen et al., 2018b	1	N/A	1	1	0	1	1	1	1	1	N/A	N/A	1	9
Houben & Jansen, 2015	1	N/A	1	1	1	1	1	1	1	1	0	0	1	10
Jansen, 2022	1	1	1	1	1	1	1	1	1	1	0	0	1	11
Kakoschke et al., 2017	1	N/A	1	1	0	1	1	1	1	1	1	1	1	11

Keeler et al., 2022	1	0	1	1	1	1	1	1	1	1	0	0	1	10
Lawrence et al., 2015a	1	0	1	1	1	1	1	1	1	1	1	1	1	12
Liu et al., 2017	1	0	1	1	0	1	1	1	1	1	0	0	1	9
Masterton et al., 2021	1	N/A	1	1	0	1	1	1	1	1	0	0	1	9
Najberg et al., 2021	1	0	1	1	1	1	1	1	1	1	1	1	1	12
Porter et al., 2021	1	N/A	1	1	0	1	1	1	1	1	0	0	1	9
Quandt et al., 2019	1	N/A	1	1	0	1	1	1	1	1	N/A	N/A	1	9
Serfas et al., 2017	1	N/A	1	1	0	1	1	1	1	1	N/A	N/A	1	9
Stice et al., 2017	1	0	1	1	1	1	1	1	1	1	0	0	1	10
Stice et al., 2021	1	0	1	1	1	1	1	1	1	1	0	0	1	10
Tzavella et al, 2021	1	N/A	1	1	1	1	1	1	1	1	N/A	N/A	1	9
Tzavella et al, 2020	1	N/A	1	1	1	1	1	1	1	1	N/A	N/A	1	9
Veling et al., 2013a Study 2	1	N/A	1	1	0	1	1	1	1	1	0	0	1	9
Yang et al., 2021a	1	0	1	1	1	1	1	1	1	1	1	1	1	12

item 1: Were subjects randomly allocated to groups (in a within-subjects design, was order randomized or counterbalanced)?

item 2[a]: Was there a description of all participants who did not complete study measures?

item 3: Were study objectives defined clearly?

item 4: Were the outcome measures defined clearly?

item 5: Was there a clear description of the inclusion and exclusion criteria?

item 6: Was there a clear description of the interventions?

item 7: Was there at least one control (comparison)
group/condition?

item 8: Were all relevant participant characteristics described? (i.e., sex, mean age)

item 9: Were complete outcome data reported (i.e., point measures and measures of
variability)?

item 10: Were outcome data reported non-selectively?

item 11[b]: Was there blinding of subjects?

item 12[b]: Was there blinding of experimenters?

item 13: Were relevant baseline measurements obtained?

[α] Single session trainings are considered as N/A.

[b] The authors gave 0 points if: the blinding of subjects or assessors was not clearly specified on the text or if the assessment of the participants' awareness of the training received was correctly guessed by ≥50% of the sample. Within study design is considered as N/A.

3. Search terms used for the meta-analysis

A. ProQuest Dissertations & Theses Global

Search terms: ab("Response inhibition" OR "Inhibitory control" OR Inhibition OR "Stop-signal" OR "Stop Signal" OR "Go/no-go" OR "No-go" OR “Executive function” OR “Executive control”) AND ti(Food OR Calorie OR Snack OR Eat OR Meat OR Liking OR Evaluation OR Craving OR Attractiveness OR Palatability) AND ab(Intervention OR Training OR Change OR Modification)

Search date: 2022/1/25; 372 Results; 3 Full texts

B. PubMed

Search terms: (("Response inhibition"[Title] OR "Inhibitory control"[Title] OR Inhibition[Title] OR "Stop-signal"[Title] OR "Stop Signal"[Title] OR "Go/no-go"[Title] OR "No-go"[Title] OR "Executive function"[Title] OR "Executive control"[Title]) AND (Food[Title/Abstract] OR Calorie[Title/Abstract] OR Snack[Title/Abstract] OR Eat[Title/Abstract] OR Meat[Title/Abstract] OR Liking[Title/Abstract] OR Evaluation[Title/Abstract] OR Craving[Title/Abstract] OR Attractiveness[Title/Abstract] OR Palatability[Title/Abstract])) AND (Intervention[Title/Abstract] OR Training[Title/Abstract] OR Change[Title/Abstract] OR Modification[Title/Abstract])

Search date: Search date: 2022/1/25; 634 Results; 37 Full texts

C. Web of Knowledge

Search Search terms: (("Response inhibition"[Abstract] OR "Inhibitory control"[Abstract] OR Inhibition[Abstract] OR "Stop-signal"[Abstract] OR "Stop Signal"[Abstract] OR "Go/no-go"[Abstract] OR "No-go"[Abstract] OR "Executive function"[Abstract] OR "Executive control"[Abstract]) AND (Food[Title] OR Calorie[Title] OR Snack[Title] OR Eat[Title] OR Meat[Title] OR Liking[Title] OR Evaluation[Title] OR Craving[Title] OR Attractiveness[Title] OR Palatability[Title])) AND (Intervention[Abstract] OR Training[Abstract] OR Change[Abstract] OR Modification[Abstract])

Search date: 2022/1/25; 3775 Results; 32 Full texts

D. PsycInfo

Search terms: ab("Response inhibition" OR "Inhibitory control" OR Inhibition OR "Stop-signal" OR "Stop Signal" OR "Go/no-go" OR "No-go" OR “Executive function” OR “Executive control”) AND ti(Food OR Calorie OR Snack OR Eat OR Meat OR Liking OR Evaluation OR Craving OR Attractiveness OR Palatability) AND ab(Intervention OR Training OR Change OR Modification)

Search date: 2022/1/25; 288 Results; 30 Full texts

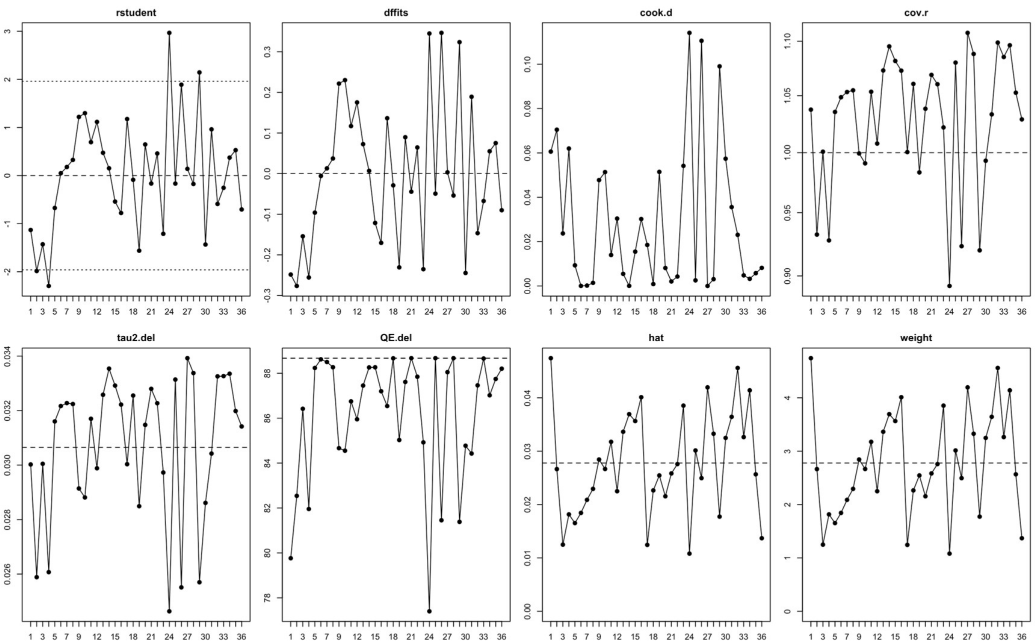

Figure S1. Plot of Influence Diagnostics. The figure shows a plot of the (1) externally standardized residuals, (2) DFFITS values, (3) Cook's distances, (4) covariance ratios, (5) leave-one-out estimates of the amount of heterogeneity, (6) leave-one-out values of the test statistics for heterogeneity, (7) hat values, and (8) weights. One study has rather large residual and may be considered outlier. However, this study actually does not have a strong influence on the results (as reflected, for example, in the Cook's distances).

Table S3. Power Analyses Describing Achieved Power to Detect Effects of Food-Specific Inhibition Training on Food Evaluation in a Two-Tailed Test.

	Achieved power to detect a		
	Small effect	Medium effect	Large effect
	(i.e., $\|g\|$ = .20)	(i.e., $\|g\|$ = .50)	(i.e., $\|g\|$ = .80)
Food-Specific Inhibition Training	1	1	1
